# Regulation of Glucose and Lipid Metabolism by Long Non-coding RNAs: Facts and Research Progress

**DOI:** 10.3389/fendo.2020.00457

**Published:** 2020-07-16

**Authors:** Tie-Ning Zhang, Wei Wang, Ni Yang, Xin-Mei Huang, Chun-Feng Liu

**Affiliations:** ^1^Department of Pediatrics, Shengjing Hospital of China Medical University, Shenyang, China; ^2^Department of Endocrinology, the Fifth People's Hospital of Shanghai, Fudan University, Shanghai, China; ^3^Department of Obstetrics and Gynecology, Yale University School of Medicine, New Haven, CT, United States

**Keywords:** lncRNA, glucose metabolism, lipid metabolism, regulatory role, tissue, cancer

## Abstract

Long non-coding RNAs (lncRNAs) are a type of non-coding RNA with a length that exceeds 200 nucleotides. Previous studies have shown that lncRNAs play an important role in the pathogenesis of various diseases. Research in both animal models and humans has begun to unravel the profound complexity of lncRNAs and demonstrated that lncRNAs exert direct effects on glucose and lipid metabolism both *in vivo* and *in vitro*. Such research has elucidated the regulatory role of lncRNAs in glucose and lipid metabolism in human disease. lncRNAs mediate glucose and lipid metabolism under physiological and pathological conditions and contribute to various metabolism disorders. This review provides an update on our understanding of the regulatory role of lncRNAs in glucose and lipid metabolism in various diseases. As our understanding of the function of lncRNAs improves, the future is promising for the development of new diagnostic biomarkers that utilize lncRNAs and treatments that target lncRNAs to improve clinical outcomes.

## Introduction

Only 1–2% of the mammalian genome is translated into proteins. Many more non-coding RNAs reside in the genome, and most of them have unknown functions (1). Long non-coding RNAs (lncRNAs) are a special type of non-coding RNA with a length that exceeds 200 nucleotides. Abundant studies have indicated that lncRNAs can have different expression patterns in disease states (2, 3). Notably, lncRNAs have been shown to mediate various biological processes in cells, including glucose and lipid metabolism (4, 5).

Glucose is the major carbon source for cellular biosynthesis and energy generation (6). Normal glucose metabolism is essential for cell life and influences cell survival. Glucose metabolism occurs through the glycolysis pathway, pentose phosphate pathway, and serine synthesis pathway in the cytoplasm and tricarboxylic acid cycle in mitochondria. Cells are well-known to acquire energy via glycolysis in the cytosol, followed by mitochondrial oxidative phosphorylation under aerobic conditions (7). When oxygen is scarce, cells rely on glycolysis rather than oxygen-consuming mitochondrial metabolism for an energy supply (7). Glucose metabolic reprogramming is widely observed during different disease states. For example, cancer cells preferentially perform glycolysis in the cytosol even in the presence of oxygen, a phenomenon that is known as the “Warburg effect” or “aerobic glycolysis.” Glucose metabolism is also related to lipid metabolism. Lipid metabolism refers to the synthesis and degradation of lipids in cells and involves the breakdown or storage of fats for energy and the synthesis of structural and functional lipids. Aberrant lipid metabolism is associated with higher concentrations of plasma lipids, including low-density lipoprotein (LDL), cholesterol, very-low-density lipoprotein, and triglycerides, and involved in such diseases as atherosclerosis (8). The regulatory network of glucose and lipid metabolism is complex. A firm understanding of the regulation of metabolism may be key to inducing alterations of the process and improve the prognosis of various types of diseases. Substantial studies have explored the regulatory process of glucose and lipid metabolism from the perspective of lncRNAs in an effort to provide new insights into the regulatory mechanism of glucose and lipid metabolism.

To enhance our understanding of lncRNAs in glucose and lipid metabolism, a comprehensive summary of the role of lncRNAs in glucose and lipid metabolism is needed. The present review provides an update on lncRNA-mediated metabolism and highlights the role of lncRNAs in various diseases that involve glucose and lipid metabolism.

## Overview of LncRNAs

lncRNAs are found in every branch of life, displaying a high degree of tissue- and cell-specific distribution (9). lncRNAs can be categorized into intergenic, intronic, bidirectional, sense, and antisense lncRNAs, depending on their genomic position relative to nearby protein-coding genes (10, 11). Notably, large intergenic non-coding RNA (lincRNAs) are transcribed from intergenic regions by RNA polymerase II; hence, they are presumably capped, polyadenylated, and spliced exactly as mRNAs (12). To date, lincRNAs are the most abundant type of lncRNAs.

**Figure 1:**
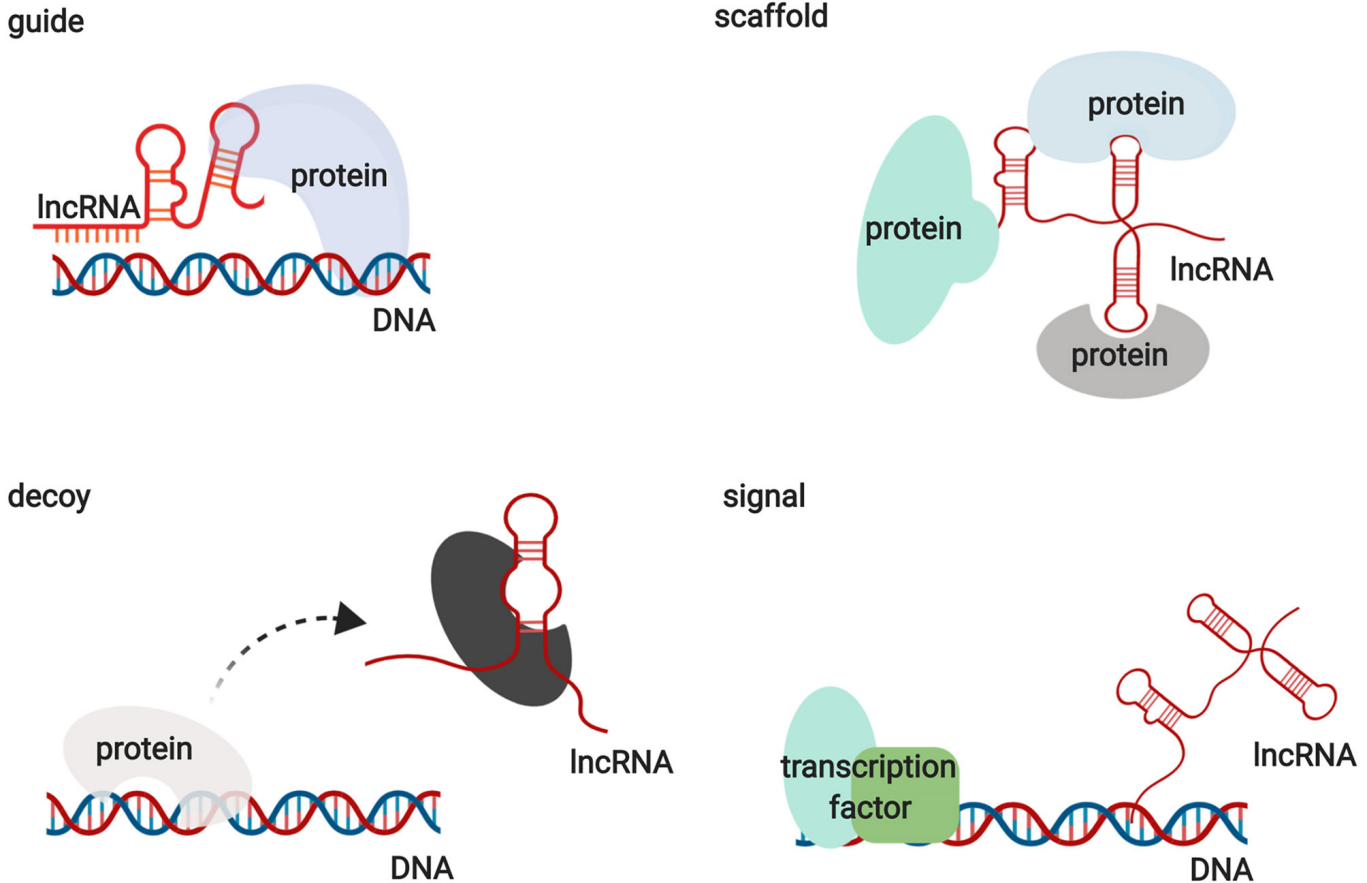
The action of lncRNAs via various mechanisms.

Functionally, lncRNAs contribute to transcriptional and post-transcriptional functions and can be broadly classified as signaling, decoy, guide, or scaffold molecules (Figure 1) (13). lncRNAs participate in regulating gene expression by interacting with proteins, RNAs, and DNAs through various mechanisms. Interestingly, the functions of lncRNAs have been shown to be associated with their unique subcellular localization (14). Many lncRNAs are recognized as nuclear modulators and have distinct nuclear localization patterns. One group of nuclear lncRNAs, lncRNA CCAT1-L, accumulates at their sites of transcription and regulate adjacent gene expression in *cis* by recruiting transcription factors, chromatin organizers, and chromatin modifiers or by forming a DNA-RNA-protein triplex (14, 15). lncRNAs that are localized in *cis*, including sno-lncRNAs, can also modulate gene expression in *trans* by acting as decoys to sequester RNA-binding proteins (RBPs) (16). Another group of lncRNAs relocates from their sites of transcription to exert functions on gene regulation in *trans* via the epigenetic control of transcription, recruiting chromatin modifiers to target genes or acting as decoys for RBPs. HOX transcript antisense RNA (HOTAIR) is one of the best examples of this group of lncRNAs (17). In addition to nuclear lncRNAs, other lncRNAs need to be exported into the cytoplasm to perform their functions. Abundant research has illustrated that cytoplasmic lncRNAs regulate gene expression by modulating signaling pathways, acting as microRNA and protein decoys, acting as scaffolds to recruit RBPs to decay target mRNAs, and controlling protein degradation pathways (18–22). These studies indicate that lncRNAs are large and diverse transcripts that participate in modulating gene expression through various mechanisms.

## Role of LncRNAs in Regulating Glucose Metabolism

### Role of lncRNAs in Glucose Metabolism Under Physiological Conditions

#### Role of lncRNAs in the Pancreas Under Physiological Conditions

**Figure 2:**
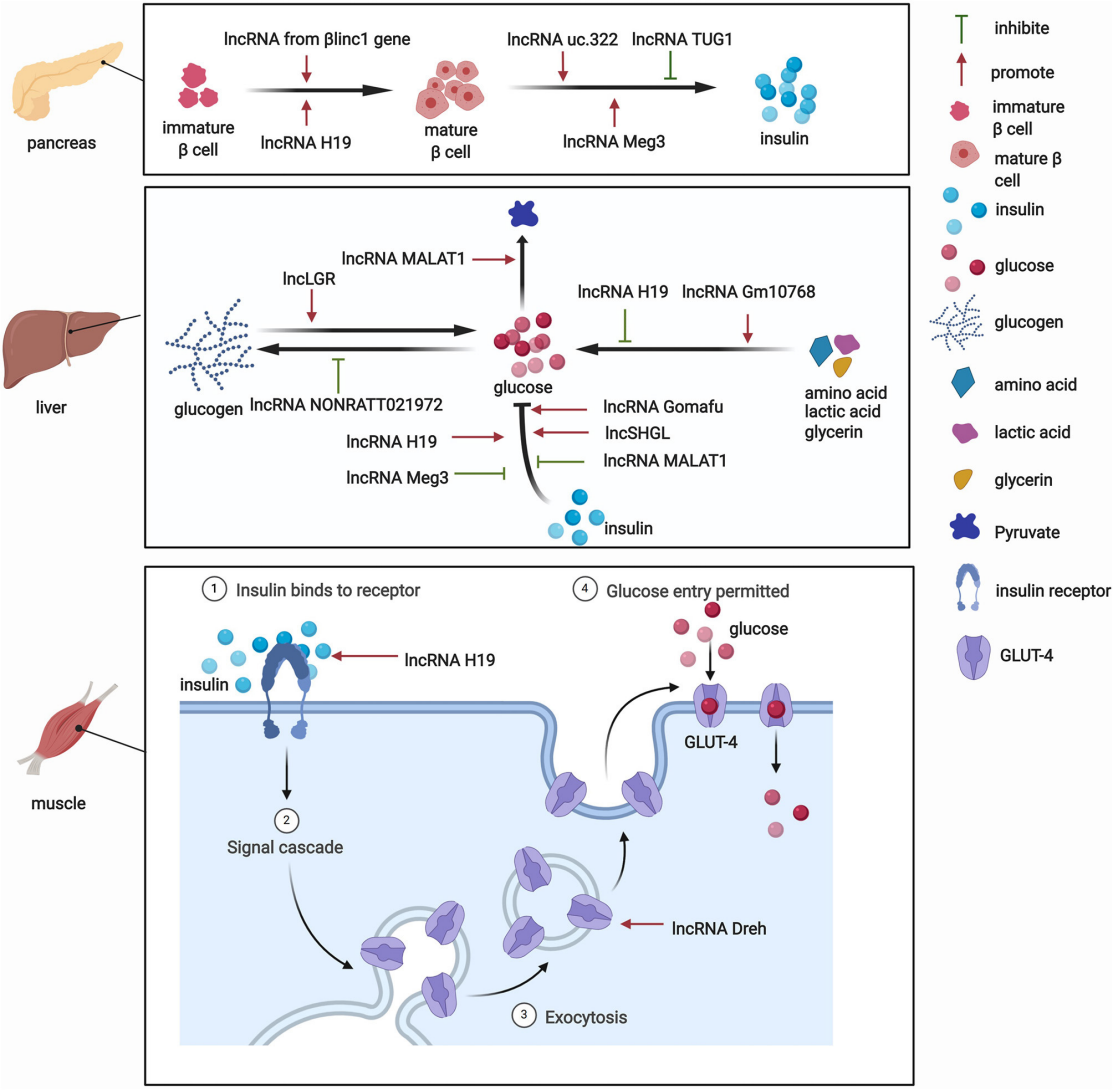
The regulatory roles of lncRNAs in glucose metabolism.

The pancreas is involved in glucose metabolism. Pancreatic β-cells maintain blood glucose levels within a physiologically relevant range by precisely adapting insulin secretion to circulating levels of nutrients (23). lncRNAs play key roles in diverse processes that occur in pancreatic β-cells (Figure 2). The expression of lncRNAs in pancreatic islet cells is often highly tissue-specific, associated with clusters of open chromatins, and located in the genomic vicinity of transcription factors that are involved in β-cell development and related functions (24–26). The development of pancreatic β-cells occurs during the neonatal period. Pancreatic β-cells expand throughout the neonatal period. Defects in this process can predispose individuals to abnormal glucose metabolism or even diabetes during adulthood. Sanchez-Parra et al. found that lncRNA H19 regulated the expansion process of pancreatic β-cells through miRNA let-7 and the Akt signaling pathway (27). After silencing lncRNA H19, the β-cell expansion process decreased in newborns, whereas the re-expression of lncRNA H19 promoted the proliferation of β-cells in adults (27). The β*linc1* gene encodes a lncRNA that can influence islet β-cell formation and function (28). Luis et al. found that β*linc1* knockout mice were glucose-intolerant and had aberrant β-cells and endocrine development (28). At the molecular level, in the absence of β*linc1*, many genes (e.g., *Neurod1*) that are involved in the specification of endocrine progenitors and maturation and function of β-cells were dysregulated (28). These studies suggest that lncRNAs participate in the regulatory process of β-cell development, but more studies need to explore the role of other lncRNAs in the development of pancreatic β-cells.

In addition to their impact on the growth of pancreatic β-cells, lncRNAs also regulate the biogenesis and secretion of insulin and thus indirectly mediate glucose metabolism. lncRNAs regulate the process of insulin biogenesis. For example, lncRNA Meg3 was shown to epigenetically regulate the expression of transcription factors, including Rad21, Smc3, and Sin3α, via EZH2-driven H3K27 methylation in pancreatic β-cells (29). By inhibiting the expression of Rad21, Smc3, and Sin3α, lncRNA Meg3 promoted the expression of MafA and promoted the synthesis of insulin both *in vivo* and *in vitro* (29). This study confirmed the role of lncRNAs in the function of islet cells by affecting insulin synthesis. lncRNAs also participate in insulin secretion. For example, Zhao et al. found that the upregulation of lncRNA ultraconserved 322 (uc.322) expression increased the expression of the insulin transcription factors pancreatic and duodenal homeobox 1 and Forkhead box O1 (FoxO1), thereby promoting insulin secretion (30). Interestingly, lncRNA Meg3 influences insulin secretion. The suppression of lncRNA Meg3 expression *in vitro* impaired insulin synthesis and secretion and increased the apoptosis rate of β cells, and these effects were mediated by the lower expression of Pdx-1 and MafA (31). Additionally, another lncRNA, TUG1, was highly expressed in pancreatic tissue compared with other organ tissues, and its expression was dynamically regulated by glucose in Nit-1 cells (32). The knockdown of lncRNA TUG1 expression increased the apoptosis ratio and decreased insulin secretion in β-cells both *in vitro* and *in vivo* (32). These changes may explain the critical role of lncRNAs in pancreatic islet cells and their influence on glucose metabolism.

#### Role of lncRNAs in the Liver Under Physiological Conditions

Besides their impact on pancreatic tissue, lncRNAs also exert functions by regulating glucose metabolism in other tissues. Various functions of the liver, particularly glucose metabolism, are performed by hepatocytes. The liver is responsible for the synthesis of glycogen via glycogenesis, the release of glucose into the blood through glycogenolysis, the breakdown of glycogen into glucose, and gluconeogenesis. Under physiological conditions, lncRNAs have functions in glucose metabolism (Figure 2). One lncRNA, hepatic glucokinase (GCK) repressor (lncLGR), can be induced by fasting. The physiological overexpression of lncLGR to mimic fasting suppressed GCK expression and reduced hepatic glycogen content in mice (33). Mechanistically, lncLGR can specifically bind to heterogenous nuclear ribonucleoprotein L, which was further confirmed to be a transcriptional repressor of GCK, thereby establishing a lncRNA-mediated mechanism that regulates hepatic GCK expression and glycogen deposition (33). This study demonstrated the essential role of lncRNAs in the regulatory process of glucose metabolism.

#### Role of lncRNAs in Other Tissues Under Physiological Conditions

lncRNAs were also shown to have functions in adipocytes under physiological conditions. For example, the lncRNA steroid receptor RNA activator 1 (SRA1) was the first functional lncRNA that was identified to regulate of adipogenesis (34). This study showed that SRA1 overexpression promoted adipogenesis by modulating PPARγ, which regulates the pro-adipogenic transcription program, and by modulating P38/c-Jun N-terminal kinase (JNK) phosphorylation *in vitro* (34). The knockdown of endogenous SRA1 inhibited 3T3-L1 preadipocyte differentiation (34), and SRA1 knockout improved insulin sensitivity and glucose tolerance (35). In addition, Zhang et al. found that the downregulation of lncRNA slincRAD expression impaired the development of adipose tissue, leading to abnormal glucose and lipid metabolism and generating a slim phenotype in both male and female mice (36). These studies highlighted a physiologically important role for lncRNAs in the development of adipocytes, which is closely associated with systemic insulin resistance.

Skeletal muscles play a pivotal role in regulating systemic glucose homeostasis. Recently, some studies have demonstrated the effects of lncRNAs on glucose metabolism in skeletal muscles (Figure 2). For example, the knockdown of the lncRNA Dreh in myotubes reduced glucose concentrations in the culture medium, increased glucose transport, and increased glucose transporter 4 (GLUT4) protein levels in C2C12 skeletal cells (37). In addition, the lncRNA H19 was shown to enhance muscle insulin sensitivity at least partially by activating the adenosine monophosphate-activated protein kinase signaling pathway, which appeared to increase glucose uptake and mitochondrial biogenesis and increase muscle insulin sensitivity (38).

### Role of lncRNAs in Glucose Metabolism Under Pathological Conditions

#### Role of lncRNAs in Glucose Metabolism in the Liver Under Pathological Conditions

Under pathological conditions, several pathways, including gluconeogenesis, are dysregulated and become core factors in different kinds of diseases (39). The dysregulation of hepatic gluconeogenesis might be attributable to alterations of the expression of gluconeogenic genes that are mediated by a complex interplay between transcription factors and other regulators, such as lncRNAs (40–42). For example, lncRNA H19 inhibition *in vivo* induced hyperglycemia and impaired glucose, insulin, and pyruvate tolerance in liver tissue. These functions of lncRNA H19 might derive from an increase in hepatic transcript levels of gluconeogenic genes and the FoxO1 gene (42). Goyal et al. also reported important functions of lncRNA H19 (43). They found that H19 depletion in HepG2 cells impaired insulin signaling and increased the nuclear localization of FoxO1 (43). Moreover, based on genome-wide methylation and transcriptome analyses, the role of H19 was highlighted in the liver. One study found that lncRNA H19 knockdown in hepatic cells altered the promoter methylation and expression of Hnf4a (a master gluconeogenic transcription factor), and this regulation was recapitulated *in vivo* (44).

In addition to lncRNA H19, some studies have explored the potential role of other lncRNAs in liver tissue. In high-fat diet-fed mice, lncRNA Meg3 was upregulated in hepatocytes (45). The knockdown of lncRNA Meg3 might mediate decreases in the expression of FoxO1 and the FoxO1 downstream targets phosphoenolpyruvate carboxykinase and the glucose-6-phosphatase catalytic subunit to improve impairments in glucose and insulin tolerance in liver tissue (46). Additionally, lncRNA Gomafu inhibited hepatic glucose production, improved insulin sensitivity, functioned as an miR-139 sponge, and led to the de-repression of its target gene FoxO1 in obese mice (47). The overexpression of hepatic lncRNA Gomafu resulted in an increase in random and fasting blood glucose levels in mice (47). The expression of a novel liver-enriched lncRNA, Gm10768, significantly increased in the liver in fasted mice and was induced by gluconeogenic hormonal stimuli (48). The knockdown of lncRNA Gm10768 improved glucose tolerance and hyperglycemia through miR-214-activating transcription factor 4 in liver tissue in diabetic db/db mice (48). Song et al. reported that lncRNA NONRATT021972 levels in the liver increased in T2DM rats, and this increase was associated with an increase in blood glucose levels (49). At the molecular level, NONRATT021972 siRNA enhanced phospho-Akt levels, hepatic glucokinase expression, and hepatic glycogen synthesis (49). In human hepatic epithelial L-02 cells, lncRNA MALAT1 acts through HIF-1α stabilization and may be a mediator that enhances arsenite-induced glycolysis (50). Based on these *in vitro* and *in vivo* studies, lncRNAs are critical in regulating glucose metabolism in liver tissue.

#### Role of lncRNAs in Glucose Metabolism in Other Tissues Under Pathological Condition

Several studies have investigated specific lncRNAs that might participate in regulating glucose metabolism in skeletal muscles under pathological condition. The recent study by Han et al. reported that a total of 144 lncRNAs, including 70 upregulated and 74 downregulated lncRNAs, were significantly differentially expressed in insulin-resistant skeletal muscle cells compared with control cells. The differentially expressed lncRNAs were significantly enriched in the peroxisome proliferator-activated receptor (PPAR) signaling pathway and insulin signaling pathway (51). Zhang et al. performed bioinformatics analyses and identified 331 lncRNAs, including 172 upregulated and 159 downregulated lncRNAs, that were differentially expressed in skeletal muscle cells in db/db mice, suggesting a critical role for the PPAR signaling pathway (52). Thus, lncRNAs have been demonstrated to participate in glucose metabolism in skeletal muscles, but the mechanisms by which this occurs are not fully understood and require further exploration.

Only a few studies have explored the association between lncRNAs and adipocytes in glucose metabolism under pathological condition. For example, Yang et al. identified a lncRNA that regulates glucose homeostasis in human adipose tissue based on a microarray assay. They found that a decrease in lncRNA uc001kfc.1 could be a *cis* regulator of phosphatase and tensin homolog (PTEN) to enhance insulin sensitivity in white adipocytes in obese patients (53). However, more research is needed to explore the influence of lncRNAs in adipocytes from the perspective of glucose metabolism.

## Role of LncRNAs in Regulating Lipid Metabolism

**Figure 3:**
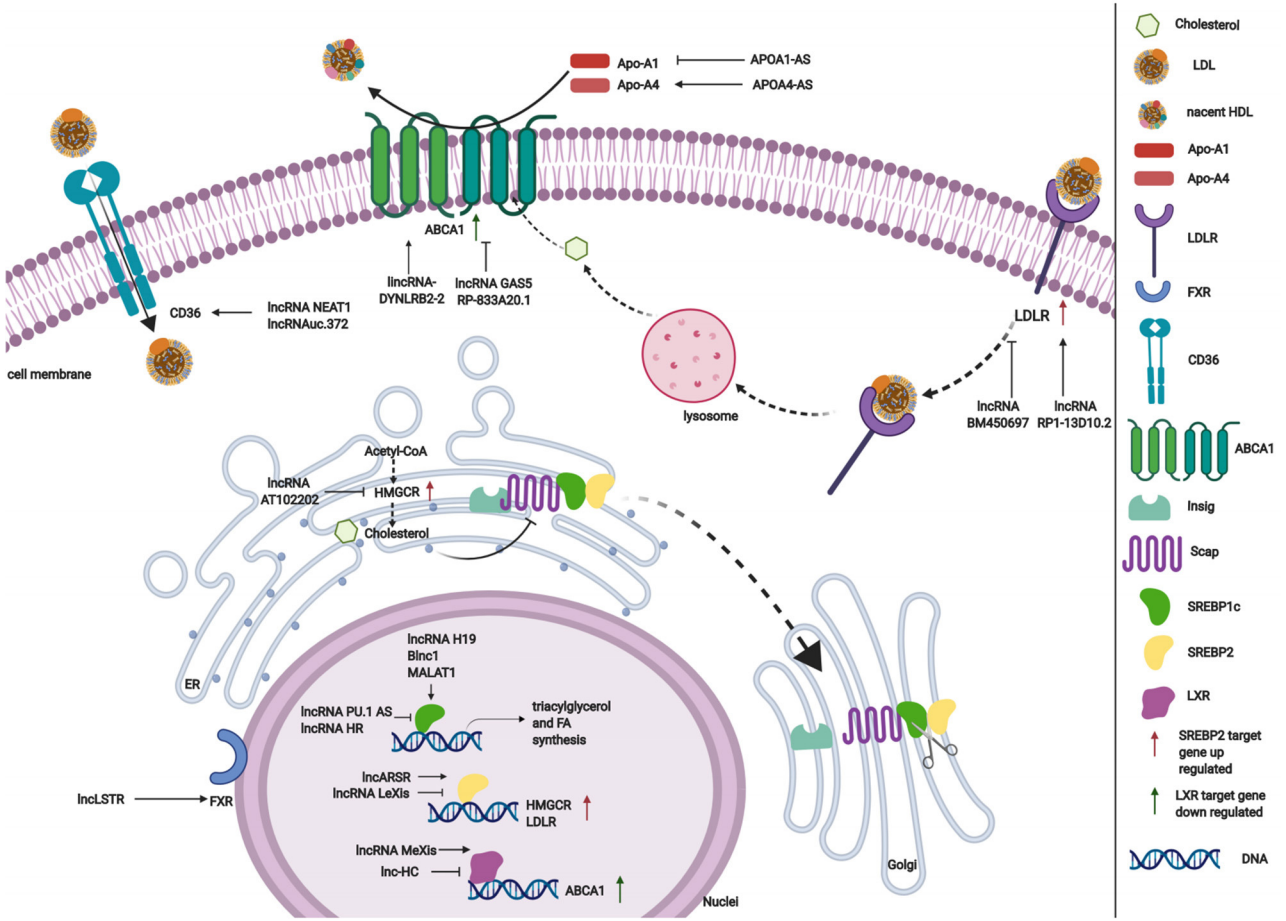
The regulatory roles of lncRNAs in lipid metabolism.

LncRNAs affect gene expression that is involved in lipid metabolism (Table 1). Accumulating studies have revealed that lncRNAs participate in lipid metabolism by influencing the expression of key genes, networks, and pathways that are involved in cholesterol and triglyceride biosynthesis, cholesterol transport, lipid uptake, and efflux (Figure 3).

**Table 1:** The effect of lncRNAs in lipid metabolism.

**Name**	**Year**	**Cell type/Model**	**lncRNA**	**Target**	**Functions in lipid metabolism**	**References**
Huang et al.	2018	C57BL6/J mice	lncARSR	SREBP-2, HMGCR	Promote cholesterol biosynthesis via PI3K/Akt/SREBP-2/HMGCR pathway	(54)
Sallam et al.	2016	Mouse primary hepatocytes and peritoneal macrophages	lncRNA LeXis	SREBP-2, HMGCR	Reduce cholesterol biosynthesis through reducing the expression of SREBF2 and HMGCR via preventing the Raly-mediated recruitment of RNA polymerase II	(55)
Liu et al.	2015	HepG2 cells	AT102202	HMGCR	Reduce cholesterol biosynthesis through downregulating HMGCR	(56)
Liu et al.	2018	Hepa1cell, mouse primary hepatocytes and mouse liver	H19	SREBP-1c	Promote triacylglycerol biosynthesis through PTBP1/SREBP-1c pathway	(57)
Zhao et al.	2018	Mouse primary hepatocytes and CRISPR/Cas9 mice	Blnc1	SREBP-1c	Promote triacylglycerol biosynthesis via binding to transcription factor EBF2	(58)
Yan et al.	2016	Livers of ob/ob mice and HepG2 cells	MALAT1	SREBP-1c	Promote triacylglycerol biosynthesis via increasing nuclear SREBP-1c protein	(59)
Dong et al.	2019	Mouse normal liver cell line and Arsenic-fed mice	PU.1 AS	SREBP-1c	Reduce triacylglycerol biosynthesis via through EZH2/Sirt6/SREBP-1c pathway	(60)
Li et al.	2017	Huh7 cells and C57BL/6J mice	lncRAN HR1	SREBP-1c and FAS	Reduce triacylglycerol biosynthesis by repressing SREBP-1c gene expression.	(61)
Lan et al.	2019	CBRH-7919 cells and E3 rat	lncHC	PPARγ	Promote triacylglycerol biosynthesis by miR-130b-3p/PPARγ pathway	(62)
Hu et al.	2014	THP-1 cells and apoE^−/−^ mice	DYNLRB2-2	ABCA1	Promote cholesterol efflux via GPR119/GLP-1R/ABCA1 pathway	(63)
Sallam et al.	2018	Mouse primary peritoneal macrophages and hepatocytes, THP-1 cells and Ldlr^−/−^ mice	MeXis	ABCA1	Promote cholesterol efflux through interacting with and guiding promoter binding of DDX17	(64)
Lan et al.	2016	CBRH-7919 cells and E3 rat	lncHC	ABCA1	Reduce cholesterol efflux through forming a complex with hnRNPA2B1 to inhibit ABCA1 and Cyp7a1	(65)
Meng et al.	2019	THP-1 cells and apoE^−/−^ mice	GAS5	ABCA1	reduce cholesterol efflux via reducing EZH2-mediated ABCA1 transcription inhibition	(66)
Hu et al.	2015	Macrophage-derived foam cells	RP5-833A20.1	miR-382, NFIA	Reduce cholesterol efflux via miR-382-5p/NFIA pathway	(67)
Halley et al.	2014	HepG2 cells and African green monkeys	APOA1-AS	APOA1	Inhibit expression of APOA1 and formation of HDL	(68)
Qin	2016	ob/ob mice	APOA4-AS	APOA4	Increase expression of APOA4	(69)
Ray	2019	HepG2 cells and human primary hepatocytes	BM450697	LDLR	Reduce lipid uptake by SREBP-1a/LDLR pathway	(70)
Mitchel et al.	2016	Huh7 and HepG2 cells	RP1-13D10.2	LDLR	Produce lipid uptake by elevating LDLR	(71)
Wang et al.	2019	THP-1 cells	NEAT1	CD36	Increase lipid uptake in macrophages by miR-342-3p/CD36 pathway	(72)
Li et al.	2015	C57BL/6 mice	lncLSTR	APOC2	Produce triglyceride clearance via TDP-43/FXR/apoC2-dependent pathway	(73)
Xu et al.	2010	3T3-L1 cells	SRA1	PPARγ	Promote white preadipocyte differentiation partly via binding to PPARγ	(34)
Liu et al.	2018	Human adipose tissue-derived mesenchymal stem cells	lncRNA TINCR	C/EBP-α	Promote adipocyte differentiation via lncRNA TINCR/miR-31-5p/C/EBP-α feedback loop	(74)
Huang et al.	2019	3T3-L1 cells	Meg3	Dickkopf-3	Promote 3T3-L1 cells preadipocyte differentiation by acting as a miR-217 sponge	(75)
Pang et al.	2013	3T3-L1 cells	PU.1AS	PU.1	Promote adipogenesis through forming an RNA duplex with PU.1 mRNA	(76)
Shang et al.	2018	Rat bone marrow mesenchymal stem cells	lncRNA TCONS_00041960	GILZ	Inhibit adipogenesis acting as a miR-125a-3p sponge	(77)
Liu et al.	2018	3T3-L1 cells	GAS5	PTEN	Inhibit adipogenesis as a miR-21a-5p sponge	(78)
Cai et al.	2018	Mouse primary preadipocytes and C57BL/6 J mice	AdipoQ AS	AdipoQ	Inhibit adipogenesis through forming an RNA duplex with AdipoQ mRNA	(79)
Schmidt et al.	2018	Primary brown adipocytes and preadipocytes from mice	H19	PEG	promote brown adipogenesis by PEG-inactivating H19-MBD1 complexes	(80)
Zhao et al.; Mi et al.; Li et al.	2014; 2016; 2017	Primary preadipocytes from mice	Blnc1	EBF2, Zbtb7b	Promote brown and beige adipocyte differentiation via binding to EBF2 or Zbtb7b	(81–83)
Alvarez-Dominguez et al.	2015	Primary brown adipocytes from mice	lncBATE1	PPARγ, C/EBPα, and C/EBPβ	Promote brown adipogenesis possibly via binding to heterogeneous nuclear ribonucleoprotein U	(84)
Bai et al.	2017	Primary preadipocytes from mice and 3T3-L1 cells	lncBATE10	Pgc1α	Promote brown adipogenesis by protecting Pgc1α mRNA from repression by Celf1	(85)
Cui et al.	2016	Primary brown adipocytes from mice	uc.417	Ucp1	Impair brown adipogenesis by inhibiting phosphorylation of p38MAPK	(86)

### Role of lncRNAs in Cholesterol and Triglyceride Biosynthesis

Cholesterol and triglyceride biosynthesis play an important role in regulating lipid metabolism. Several studies have shown that lncRNAs participate in expression of the transcription factor SREBP, which controls the expression of numerous enzymes that are required for endogenous cholesterol, fatty acid (FA), triacylglycerol, and phospholipid synthesis (59, 87, 88). The SREBP family is divided into three isoforms (SREBP-1a, SREBP-1c, and SREBP-2) that preferentially activate the transcription of cholesterol biosynthesis genes (88). Huang et al. showed that lncARSR overexpression increased the expression of 3-hydroxy-3-methyl-glutaryl-coenzyme A reductase (HMGCR), the rate-limiting enzyme of cholesterol synthesis, and promoted hepatic cholesterol biosynthesis *in vivo*. Mechanistically, lncARSR activates the phosphatidyl inositol 3-kinase (PI3K)/Akt pathway, thereby increasing the expression of mature SREBP-2, which is a primary transcription factor of HMGCR (54). In contrast to lncARSR, several other lncRNAs play negative regulatory roles in cholesterol biosynthesis. For example, lncRNA LeXis expression increased in the liver when mice were fed a Western diet and when liver X receptors (LXRs) were pharmacologically activated, thus demonstrating that lncRNA LeXis functioned as a modulator of genes that are involved in cholesterol homeostasis. Activated LeXis in chow-fed mice reduced the expression of cholesterol biosynthesis genes, including SREBF2 and HMGCR, and decreased serum and hepatic cholesterol levels. LeXis was also shown to interact with the heterogeneous ribonucleoprotein RALY and prevented the Raly-mediated recruitment of RNA polymerase II to promoters of cholesterol biosynthesis genes (55). Microarray analysis showed that another lncRNA, AT102202, influenced HMGCR in HepG2 cells, and AT102202 knockdown resulted in an increase in HMGCR expression in HepG2 cells (56).

Unlike SREBP-2, lncRNA-mediated SREBP-1c preferentially enhances the transcription of triacylglycerol and FA synthesis genes (88). Liu et al. showed that H19 expression was upregulated by FAs in hepatocytes and in diet-induced fatty liver, which further augmented triacylglycerol accumulation (57). At the molecular level, they found that H19 could interact with polypyrimidine tract-binding protein 1 (PTBP1) to facilitate its association with sterol regulatory element-binding protein 1c mRNA and protein, leading to increased stability and nuclear transcriptional activity (57). Wang et al. studied lncRNA H19-mediated SREBP and found that H19 expression increased in oleic acid-induced steatosis and during the development of HFD-induced NAFLD. H19 activation induced lipid accumulation by upregulating both the MLXIPL transcriptional network and the mTORC1 signaling axis (including active SREBP) *in vivo* and *in vitro* (89). Brown fat lncRNA 1 (Blnc1) has been shown to regulate adipocyte differentiation (81). It may also serve as a regulator of triacylglycerol biosynthesis. Zhao et al. showed that the overexpression of Blnc1 in primary hepatocytes significantly augmented the induction of SREBP1c, increased the expression of triacylglycerol biosynthesis genes, and induced hepatic steatosis and insulin resistance through LXR activation. At the molecular level, proteomic analysis of the Blnc1 ribonucleoprotein complex identified EDF1 as a component of the LXR transcriptional complex that acted in concert with Blnc1 to activate the lipogenic gene program (58). Yan et al. found that the palmitate-mediated augmentation of MALAT1 expression occurred concomitantly with an increase in SREBP1c expression in hepatic cells. MALAT1 knockdown decreased the palmitate-induced lipid accumulation and expression of nuclear SREBP-1c protein in HepG2 cells and primary mouse hepatocytes by reducing the binding of MALAT1 to SREBP-1c in the nucleus, thereby inhibiting its ubiquitination to stabilize SREBP-1c protein (59). Several lncRNAs are also involved in the transcriptional suppression of SREBP-1c expression (60, 61). For example, lncRNA PU.1 AS expression was significantly upregulated in the liver in chronic arsenic-feed mice, which exhibited a decrease in serum triglyceride levels (60). At the molecular level, PU.1 AS was shown to modulate triacylglycerol homeostasis by interacting with EZH2 protein to inhibit sirtuin 6 mRNA and protein expression, thereby decreasing the expression of SREBP-1c and lipid accumulation (60). Similar to PU.1 AS, lncRNA HR functions as a negative regulator of SREBP1c in the mouse liver (61). *In vitro* and *in vivo*, lncHR1 overexpression inhibited the activation of SREBP-1c and FAS and decreased triglyceride synthesis and lipid droplet formation in oleic acid-induced hepatic cells and high-fat diet-fed mice (61). In addition to the influence of lncRNAs on SREBP-1c, a reduction of lncHC expression increased triacylglycerol and FA biosynthesis by decreasing miR-130b-3p expression, thereby inducing PPARγ activation (62).

### Role of lncRNAs in Lipid Efflux

Lipid efflux and reverse cholesterol transport are important steps in lipid homeostasis, playing important roles in removing and transporting excess lipids and cholesterol from intracellular stores. Abca1 is a critical cholesterol efflux gene that encodes the plasma membrane transporter ATP-binding cassette transporter ABCA1, which transports excess cellular cholesterol from cells to its corresponding apolipoproteins (90). Several studies have explored the relationship between ABCA1 and lncRNAs (63, 66). Hu et al. found that oxidized LDL (ox-LDL) significantly induced lincRNA DYNLRB2-2 expression, which promoted ABCA1-mediated cholesterol efflux and inhibited inflammation through G protein-coupled receptor 119 in THP-1 macrophage-derived foam cells (63). Similar to lincRNA DYNLRB2-2, lncRNA MeXis was shown to be upregulated in ox-LDL and LXR (GW3965) agonist-treated macrophages, which correlated with cellular lipid content. MeXis knockout mice exhibited a reduction of Abca1 expression, and a loss-of-function of MeXis in mouse bone marrow cells altered chromosome architecture at the Abca1 locus, impaired macrophage cholesterol efflux, and accelerated the development of atherosclerosis. MeXis was also shown to interact with and guide promoter binding of the transcriptional coactivator DDX17, followed by amplification of the LXR-dependent transcription of Abca1 in macrophages (64). In contrast to the positive regulation of LXRs by MeXis, lncHC has antagonistic effects on LXR activity, demonstrating its function as a negative regulator of cholesterol metabolism both *in vitro* and *in vivo* (65). Mechanistic studies indicated that lncHC increased the expression of hnRNPA2B1 in the nucleus and formed an RNA-protein complex, which further bound to cholesterol 7α-hydroxylase (Cyp7a1) and Abca1 mRNAs and reduced their expression (91). Another lncRNA that has a negative effect on ABCA1 is growth arrest-specific 5 (GAS5) (66). A recent study showed that lncRNA GAS5 was mainly localized in the nucleus and highly expressed in THP-1 macrophage-derived foam cells in coronary heart disease. The knockdown of GAS5 increased cholesterol efflux and inhibited intracellular lipid accumulation in THP-1 macrophage-derived foam cells and homozygous apolipoprotein E (ApoE) knockout mice by reducing the EZH2-mediated transcriptional inhibition of ABCA1 by histone methylation (66). The overexpression of RP-833A20.1 in THP-1 macrophages may also decrease cholesterol efflux. RP5-833A20.1 appeared to exert its effects on cholesterol content in macrophages by increasing miR-382 levels to downregulate nuclear factor IA (NFIA) (67).

Lipoprotein formation is also important in regulating lipid metabolism by binding and transporting lipids to various tissues in the body for metabolism. Two AS lncRNAs, APOA1-AS and APOA4-AS, have been reported to regulate the expression of apolipoproteins that contribute to the formation or function of plasma lipoproteins (68, 69). Halley et al. identified APOA1-AS as a negative transcriptional regulator of APOA1, which is the major protein component of high-density lipoprotein (HDL) in plasma and plays a key role in reverse cholesterol transport both *in vitro* and *in vivo*. The inhibition APOA1-AS promoted ApoA1 gene expression, likely through the recruitment of suppressor of zeste 12 homolog and the histone-modifying enzyme lysine-specific demethylase 1 to the ApoA1 promoter (68). In contrast to APOA1-AS, APOA4-AS was found to be a concordant regulator of APOA4 expression, which is a major component of HDL and triglyceride-rich lipoprotein particles and controls liver triglyceride secretion (69). The expression of APOA4-AS and APOA4 was abnormally upregulated in ob/ob mice and patients with hepatic steatohepatitis. Mechanistic experiments showed that APOA4-AS knockdown reduced APOA4 expression, leading to a decrease in serum triglyceride content and total cholesterol in ob/ob mice by directly interacting with HuR and stabilizing APOA4 mRNA (69).

### Role of lncRNAs in Lipid Uptake and Excretion

Low-density lipoprotein receptors (LDLRs) play a vital role in ingesting and removing LDL particles from the blood. Ray et al. reported that lncRNA BM450697 acted as a regulator of LDLRs. The silencing of lncRNA BM450697 resulted in the activation of LDLRs (70). At the molecular level, lncRNA BM450697 decreased LDLR mRNA levels, which was likely attributable to the blockade of interactions with RNA polymerase II and possibly SREBP1a at the LDLR promoter (70). Unlike BM450697, lncRNA RP1-13D10.2 was shown to activate the transcription of LDLRs, but the mechanisms are unclear and require further exploration (71).

When lipids accumulate in the blood, LDL undergoes oxidation modifications so that it can be ingested by CD36 and scavenger receptors. Wang et al. found that NEAT1 expression significantly increased in THP-1 cells that were treated with ox-LDL, and miR-342-3p expression significantly decreased. The silencing of NEAT1 in THP-1 cells inhibited CD36 mRNA expression and decreased Oil-Red staining levels, total cholesterol, and triglyceride content through the modulation of miR-342-3p (72). Another lncRNA that affects CD36 is uc.372 (92). uc.372 expression of genes that are involved in lipid uptake and synthesis, including CD36, ACC, FAS, and SCD1 (93).

Excess lipid excretion depends on bile acid metabolism, which is mediated by nuclear farnesoid X receptors that regulate genes that are involved in bile acid synthesis, including sterol 12α-hydroxylase (Cyp8b1) and Cyp7a1 (94). Li et al. found that a lncRNA, liver-specific triglyceride regulator (lncLSTR), in mice had an essential physiological function in mammalian lipid homeostasis via the bile acid pathway. They found that the liver-specific knockdown of lncLSTR in mice markedly reduced plasma triglyceride levels. They also found that lncLSTR knockdown decreased the binding with TDP-43, inhibited Cyp8b1 gene expression, and increased the ratio of muricholic acid to cholic acid in bile acid. Changes in bile acid composition activated FXRs to increase apolipoprotein C2 levels, resulting in greater triglyceride clearance in mice (73).

### Role of lncRNAs in Regulating Lipid Metabolism in Adipogenesis

Adipogenesis is the process by which preadipocytes develop into mature white, brown, or beige adipocytes, contributing to both lipid storage and clearance, which are associated with lipid metabolism. Emerging evidence indicates the importance of lncRNAs in adipogenesis (Table 1). For example, Qi et al. found significant alterations of the expression of 114 lncRNAs in adipogenic cells during the adipogenic *trans*-differentiation of myoblasts (95). They also found that the lncRNA GM43652 had the highest expression levels in adipogenic cells. Knockdown of GM43652 negatively influenced lipid deposition in *trans*-differentiated myoblasts (95). Additionally, the transcriptomic analysis of primary preadipocytes, brown and white adipocytes, and cultured adipocytes during adipogenesis showed that the expression of 175 lncRNAs was significantly altered during the differentiation of adipocytes (96). These findings indicate that lncRNAs play vital roles in adipogenesis.

#### Role of lncRNAs in White Adipocyte Adipogenesis

White adipocytes are derived from white adipose tissue and control the storage of triacylglycerol. As mentioned above, many lncRNAs have been shown to be related to adipogenesis and might be bound at their promoters by key transcription factors, such as PPARγ and CCAAT/enhancer-binding protein α (C/EBPα) (96). For example, the lncRNA SRA1 has been demonstrated to promote preadipocyte differentiation partly via binding to PPARγ (34). Liu et al. found that terminal differentiation-induced ncRNA (TINCR) modulated adipogenic differentiation in human adipose tissue-derived mesenchymal stem cells by serving as a ceRNA for miR-31 to target C/EBP-α (74). Similar to lncRNA TINCR, Huang et al. found that lncRNA Meg3 might induce the differentiation of 3T3-L1 preadipocytes by acting as a miR-217 sponge (75). Furthermore, antisense lncRNAs, such as PU.1AS, have been shown to be involved in adipogenesis (76). PU.1AS might regulate adipogenesis by forming an RNA duplex with PU.1 mRNA and inhibiting PU.1 mRNA translation (76). In addition to the above lncRNAs, the potential role of other lncRNAs in adipogenesis, including GM43652, GM15290, ADINR, Paral1, and NEAT1 have also been shown to be positively related to adipogenesis (95, 97–100).

In contrast to the functions of lncRNAs that are described above, the lncRNAs TCONS_00041960 and lncRNA GAS5 act as sponges of microRNAs to negatively mediate adipogenesis (77, 78). At the molecular level, Shang et al. found that TCONS_00041960 inhibited the adipogenesis of rat bone marrow mesenchymal stem cells by acting as a competing endogenous RNA that interacted with miR-125a-3p to regulate GILZ, which inhibits the adipogenic regulator PPARγ and blocks adipocyte differentiation (77). GAS5 plays a suppressive role in the adipogenesis of 3T3-L1 cells through an miRNA-based regulatory mechanism and might indirectly improve the expression of PTEN by repressing miR-21a-5p (78). An antisense lncRNA, adiponectin AS, was shown to inhibit adipogenesis by transferring from the nucleus to the cytoplasm and forming an AdipoQ AS lncRNA/AdipoQ mRNA duplex to suppress adiponectin mRNA translation in mouse primary preadipocytes and adipose tissues from high-fat-diet-fed mice (79). Other lncRNAs, such as U90926, MIR221HG, and RP11-20G13.3, have also been shown to be negatively related to adipogenesis (101–103). These studies indicate that lncRNAs regulate white adipogenesis through lncRNA-protein, lncRNA-mRNA, and lncRNA-miRNA interactions.

#### Role of lncRNAs in Brown and Beige Adipocyte Differentiation

Brown adipose tissue (BAT) and beige fat are responsible for thermogenesis. They are governed by a network of transcription factors and cofactors, including PR domain-containing 16 (PRDM16), PPARγ, PPARγ coactivator 1α (PGC-1α), early B-cell factor 2 (EBF2), and C/EBP β (104, 105). Accumulating studies have shown that several lncRNAs regulate thermogenic adipocyte biology by interfacing with these transcriptional regulators.

The function of maternally expressed, imprinted lncRNA H19 in brown adipocyte differentiation was studied by Schmidt et al. (80). Their results indicated that H19 overexpression promoted adipogenesis, oxidative metabolism, and mitochondrial respiration in brown but not white adipocytes. Mechanistically, H19 recruits paternally expressed gene (PEG)-inactivating H19-MBD1 complexes and acts as a BAT-selective PEG gatekeeper (80). Additionally, other transcripts, including Blnc1, dPrdm16, BATE1, and BATE10, have relevant functions in brown and beige adipocyte differentiation (81–85, 106). Chromatin immunoprecipitation with massively parallel DNA sequencing (ChIP-seq) in adipose tissues and during brown adipocyte differentiation showed that the expression of Blnc1 and transcriptional regulators of brown fat development (e.g., EBF2, Ucp1, and PPARγ) was high during brown adipocyte differentiation (81). Further experiments elucidated the molecular mechanisms by which Blnc1 formed a ribonucleoprotein complex with heterogeneous nuclear ribonucleoprotein U (hnRNPU) and EBF2 or zinc finger and BTB domain-containing 7b to promote the thermogenic gene program and brown and beige adipocyte differentiation (81–83). A study of *de novo* reconstruction of the human adipose transcriptome showed that 909 lncRNAs were specifically detected in BAT, and 169 conserved human lncRNAs regulated their nearby mRNAs (106). Notably, a functional lncRNA, lncdPrdm16, was shown to regulate adipogenesis *in vitro* and *in vivo* (106). To systematically study BAT-specific lncRNAs that are associated with adipogenesis in brown fat, Alvarez-Dominguez et al. reconstructed *de novo* transcriptomes of mouse brown fat, inguinal white fat, and epididymal white fat using RNA-seq and identified 127 BAT-restricted loci that were induced during differentiation (84). Among these lncRNAs, lncBATE1 was enriched in BAT compared with other organs. The silencing of lncBATE1 significantly reduced BAT-selective gene expression in primary brown adipocytes, which was partially rescued by exogenous lncBATE1 with mutated siRNA-targeting sites through an interaction with hnRNPU (84). lncBATE10, another BAT-enriched lncRNA, was required for full brown fat differentiation and white fat browning program by decoying Celf1 from PGC-1α, thereby protecting PGC-1α mRNA from repression by Celf1 (85).

Contrary to the functions of lncRNAs that are described above, several lncRNAs may act as negative regulators of brown and beige adipocyte differentiation (86, 107). For example, Cui et al. showed that the ectopic expression of uc.417 impaired adipogenesis and the thermogenic program in brown adipocytes by moderately inhibiting the phosphorylation of p38 mitogen-activated protein kinase, which is essential for BAT activation (86). These data suggest that lncRNAs control brown and beige adipocyte differentiation through lncRNA-protein interactions.

## Role and Clinical Significance of LncRNAs for Glucose and Lipid Metabolism-Related Disease

### Effect and Mechanism of Action of lncRNAs in Cancer

Cancer is a major public health problem worldwide. A recent study estimated the number of new invasive cancer cases in the United States in 2020, with approximately 1,806,590 diagnoses, equating to approximately 4,950 new cases each day (108). However, the mechanisms of cancer development are still unclear. Glucose metabolism consists of many steps and allows cells to access energy and transiently store it in adenosine triphosphate (ATP), which is necessary for the development of cancer. Some studies have suggested that lncRNAs may participate in the regulation of various cancers through effect on glucose metabolism (Table 2).

**Table 2:** The effect of lncRNAs on glucose metabolism in cancers.

**Name**	**Year**	**Cancer**	**lncRNA**	**Effect on glucose metabolism**	**References**
Zheng et al.	2019	Hepatocellular carcinoma	LINC01554	Inhibit Warburg effect via degradation of PKM2 and inhibiting the Akt/mTOR signaling pathway	(109)
Malakar et al.	2019	Hepatocellular carcinoma	lncRNA MALAT1	Promote glycolysis and inhibit gluconeogenesis by Enhancing mTOR-Mediated Translation of TCF7L2	(110)
Tang et al.	2019	Colorectal cancer	lncRNA GLCC1	Increase glycolytic metabolism by stabilizing c-Myc from ubiquitination	(111)
Feng et al.	2019	Colorectal cancer	LINC00504	Increase lactate production, glucose uptake, and pentose phosphate pathway	(112)
Li et al.	2019	Esophageal cancer	LINC00184	Promote glycolysis by enhancing the promoter methylation of PTEN	(113)
Ma et al.	2019	Pancreatic adenocarcinoma	lncRNA HOTAIR	Increase lactate production, glucose uptake and ATP production by upregulating HK-2	(114)
Chen et al.	2019	Osteosarcoma	lncRNA HAND2-AS1	Inhibit glucose uptake	(115)
Chu et al.	2019	Oral squamous cell carcinoma	LncRNA ELF3-AS1	Increase glucose metabolism by upregulating GLUT1	(116)
Cheng et al.	2019	Glioblastoma	lncRNA-XIST	Promote glucose metabolism by acting as a miR-126 sponge	(117)
Li et al.	2018	Hepatocellular carcinoma	lncRNA Ftx	Increase Warburg effect by influencing the PPAR signaling pathway	(118)
Kang et al.	2018	Osteosarcoma	lncRNA HAND2-AS1	Inhibit glucose uptake and lactate production	(119)
Han et al.	2018	Osteosarcoma	lncRNA TUG1	Increase glucose consumption and lactate production by upregulating HK-2	(120)
Sun et al.	2018	Acute myeloid leukemia	lncRNA ANRIL	Increase glucose uptake by inhibiting AdipoR1-AMPK/SIRT1 signaling pathway	(121)
Zhang et al.	2018	Acute myeloid leukemia	lncRNA UCA1	Promote glycolysis through the microRNA-125a/HK-2 pathway	(122)
Yang et al.	2018	Multiple myeloma	lncRNA PDIA3P	Increase G6PD expression and pentose phosphate pathway	(123)
Wang et al.	2018	Oral squamous cell carcinoma	lnc-p23154	Promote glycolysis by facilitating GLUT1 expression and inhibiting miR-378a-3p transcription	(124)
Xing et al.	2018	Breast cancer	LINC00538 (YIYA)	Promote glycolysis by regulating CDK6-dependent phosphorylation of the fructose bisphosphatase PFK2	(91)
Luan et al.	2018	Malignant melanoma	lncRNA H19	Promote glucose metabolism by acting as a miR-106a-5p sponge	(125)
Wei et al.	2017	Hepatocellular carcinoma	lncRNA HOTAIR	Promote glycolysis via mTOR/GLU1 pathway	(126)
Song et al.	2017	Osteosarcoma	lncRNA Pvt1	Increase glucose uptake and lactate production by regulating miR-497/HK2 axis	(127)
Hu et al.	2017	Bladder cancer	lncRNA CASC8	Inhibit glycolysis by interacting with FGFR1	(128)
Rupaimoole et al.	2016	Ovarian cancer	lncRNA NRCP	Promote glycolysis by activating STAT1 transcriptional gene network	(129)
Li et al.	2014	Bladder cancer	lncRNA UCA1	Promote glycolysis through the mTOR-STAT3/microRNA143 pathway	(130)

#### Tumors of the Digestive System

lncRNAs may participate in regulating glucose metabolism in different cancers of the digestive cancers. Hepatocellular carcinoma (HCC) is a leading cause of cancer-related morbidity and mortality, with the fifth highest cancer incidence in men and the ninth in women (131, 132). Several studies have explored the potential role of lncRNAs in regulating glucose metabolism in clinical HCC patients. For example, Zheng et al. tested 167 primary HCC samples and found that LINC01554 was frequently downregulated in patients with HCC (109). At the molecular level, LINC01554 may promote the ubiquitin-mediated degradation of PKM2 and inhibit the Akt/mTOR signaling pathway to abolish the Warburg effect *in vitro* (109). Another study on 73 HCC patients and found that the lncRNA Ftx was upregulated in HCC samples and may promote the Warburg effect by influencing the PPAR signaling pathway (118). Wei et al. reported a marked increase in lncRNA HOTAIR expression in 84 HCC tissues (126). This lncRNA was shown to promote glycolysis by upregulating GLUT1 and activating mTOR signaling in HCC cells, whereas its knockdown suppressed this effect (126). In addition, the lncRNA MALAT1 was found to influence the development of HCC by regulating glucose metabolism, enhancing glycolysis, and inhibiting gluconeogenesis through elevating translation of the transcription factor TCF7L2 (110). Enhancement of translation of the lncRNA MALAT1 by TCF7L2 was also shown to be mediated by the upregulation of serine/arginine-rich splicing factor 1 (SRSF1) and activation of the mTOR complex 1 (mTORC1)–eukaryotic translation initiation factor 4E-binding protein 1 (4EBP1) axis (110). These studies demonstrate that lncRNAs regulate glucose metabolism in HCC and are potential therapeutic targets for the disease.

Colorectal cancer (CRC) kills nearly 700,000 people annually and has become the world's fourth deadliest cancer (133). Several studies have suggested a potential role for lncRNAs in glucose metabolism in CRC. Tang et al. evaluated 95 patients with CRC and found that the lncRNA GLCC1 was significantly upregulated compared with that in healthy controls. This lncRNA may stabilize the transcription factor c-Myc from ubiquitination by direct interaction with the HSP90 chaperon. The transcriptional modification pattern of c-Myc target genes, such as LDHA, consequently reprogrammed glycolytic metabolism toward CRC cell proliferation (111). In addition, LINC00504 was shown to be upregulated in CRC patients to promote colon cancer progression. This lncRNA might be a transcriptional regulator of c-Myc that reprograms central metabolism, including glucose metabolism, the pentose phosphate pathway, and the tricarboxylic acid cycle (112). These studies strongly suggest the potential influence of lncRNAs on glucose metabolism in CRC, but further studies are needed to explore the detailed mechanisms.

In other cancers of the digestive system, such as esophageal cancer (EC) and pancreatic cancer, lncRNAs participate in the pathophysiological process by influencing glucose metabolism. One study on 84 patients with EC and found that LINC00184 was upregulated in EC tissues and may mediate glycolysis (113). At the molecular level, LINC00184 was shown to enhance the promoter methylation of PTEN by recruiting DNA (cytosine-5)-methyltransferase 1 (DNMT1), and the inhibition of PTEN promoter methylation suppressed EC cell glycolysis and improved mitochondrial oxidative phosphorylation (113). Additionally, one study revealed that the lncRNA HOTAIR was upregulated in pancreatic adenocarcinoma compared with the level in adjacent healthy tissues (114). Overexpression of the lncRNA HOTAIR increased tumor cell proliferation, lactate production, glucose uptake, and ATP production in pancreatic adenocarcinoma by upregulating hexokinase-2 (HK-2) (114). These findings provide new insights into the role of lncRNAs in cancers of the digestive system.

#### Bone Tumors

Osteosarcoma is a primary malignant bone tumor that most commonly affects children, adolescents, and young adults (134). However, the mechanism behind its development remains unclear and needs further investigation. Recent studies of osteosarcoma found that lncRNAs that potentially participate in glucose metabolism may influence tumor development in this condition. Chen et al. reported that the expression of the lncRNA HAND2-AS1 decreased in osteosarcoma tissues compared with that in healthy tissues (115). Knockdown of this lncRNA promoted osteosarcoma cell proliferation, increased glucose uptake, and upregulated GLUT1 expression (115). Interestingly, Kang et al. also explored the role of this lncRNA in osteosarcoma (119). They found that it was significantly upregulated in osteosarcoma cells compared with the level in normal cultured cells, and that its knockdown increased glucose uptake, lactate production, and the expression of a series of enzymes that are involved in energy metabolism, thus influencing the development of osteosarcoma (119). Based on these studies, HAND2-AS1 appears to be involved in osteosarcoma by influencing glucose metabolism, but its precise role and molecular mechanisms need further investigation.

The lncRNA TUG1 was shown to be overexpressed in osteosarcoma cells compared with the level in a normal osteoblastic cell line (120). Its knockdown inhibited glucose consumption, lactate production, and cell viability in osteosarcoma cells (120). At the molecular level, the aberrant expression of lncRNA TUG1 markedly affected the expression of HK-2 (120). Additionally, Song et al. reported that the expression of the lncRNA Pvt1 was increased specifically in osteosarcoma cells and tissues, and its upregulation of lncRNA Pvt1 was associated with a poor prognosis (127). Its overexpression increased glucose uptake, lactate production, and the expression of HK-2 in osteosarcoma cells, while its knockdown produced the opposite effects (127). These studies highlight the importance of lncRNAs in the development of osteosarcoma through the regulation of glucose metabolism, but the exact regulatory mechanisms that underlie the relationship between lncRNAs and osteosarcoma need further study.

#### Genitourinary Malignancy

Bladder cancer is the second most common genitourinary malignancy. There has been little progress in treatments for bladder cancer, and the mechanisms by which bladder cancer develops and progresses need further investigation. A recent study reported that the lncRNA CASC8 was significantly downregulated and associated with advanced-stage bladder cancer (128). Its overexpression was shown to significantly suppressed bladder cancer cell proliferation by reducing the glycolysis of bladder cancer cells and interacting with fibroblast growth factor receptor 1 (FGFR1) (128). Another lncRNA of the UCA1 gene, which was highly expressed in bladder carcinoma and enhanced the tumorigenic behavior of bladder cancer cells *in vitro* and *in vivo* (135, 136), promoted glycolysis through the mTOR-STAT3/microRNA143 pathway in bladder cancer cells, and UCA1-induced HK-2 was shown to be an important mediator in this process (130).

In addition to bladder cancer, lncRNAs also play roles in ovarian cancer, which is the most frequent cause of death among gynecologic malignancies. For example, the lncRNA NRCP (ceruloplasmin) was found to be highly upregulated in ovarian cancer. NRCP knockdown resulted in a significant increase in apoptosis, a decrease in cell proliferation, and a decrease in glycolysis compared with the levels in control cancer cells (129). NRCP is also an intermediate binding partner between STAT1 and RNA polymerase II and can lead to an increase in the expression of downstream target genes, such as glucose-6-phosphate isomerase (129). These findings suggest the potential role of lncRNAs in glucose metabolism in ovarian cancer.

#### Malignant Hemopathy

Acute myeloid leukemia (AML) is the most common acute leukemia in adults, accounting for ~80% of leukemia cases (137). Sun et al. found that the lncRNA ANRIL was upregulated in AML patients at diagnosis and downregulated in patients after complete remission. At the molecular level, the knockdown of ANRIL expression resulted in a decrease in glucose uptake and the inhibition of AML cell maintenance through the targeting of adiponectin receptors *in vitro* and *in vivo* (121). Knockdown of the lncRNA UCA1 was shown to play a beneficial role in overcoming chemoresistance in pediatric AML (122). A functional study showed that the knockdown of this lncRNA potentiated the cytotoxic effect of adriamycin (ADR) and inhibited HIF-1α-dependent glycolysis in ADR-resistant AML cells (122).

Multiple myeloma (MM) is the second most common hematologic malignancy. lncRNAs also regulate glucose metabolism in MM. For example, Yang et al. showed that the lncRNA PDIA3P was highly expressed in MM and associated with survival rate in MM patients (123). This lncRNA may also interact with c-Myc to enhance its transactivation and binding to the glucose 6-phosphate dehydrogenase (G6PD) promoter, stimulating G6PD expression and pentose phosphate pathway flux (123). This study elucidated crucial roles for lncRNAs in the regulation of glucose metabolism in MM, providing a potential therapeutic target for this disease.

#### Other Cancers

Some studies have suggested that lncRNAs may be involved in glucose metabolism in other kinds of cancer. For example, a study of 112 patients with oral squamous cell carcinoma (OSCC) reported that the lncRNA ELF3-AS1 was upregulated and positively correlated with GLUT1. This lncRNA was shown to positively regulate OSCC cell proliferation through GLUT1 and reprogrammed glucose metabolism *in vitro* (116). In addition, a novel lncRNA, lnc-p23154, was upregulated in OSCC and affected glycolysis by facilitating GLUT1 expression and inhibiting miR-378a-3p transcription (124). These recent studies highlight the modulatory role of lncRNAs in glucose metabolism in OSCC.

In addition, in the case of breast cancer, the lncRNA LINC00538 (YIYA) was expressed in ~40% of cases and correlated with cyclin-dependent kinase 6 (CDK6) expression and unfavorable survival outcomes (91). Its upregulation promoted glycolysis, was associated with cytosolic CDK6, and regulated the CDK6-dependent phosphorylation of fructose bisphosphatase PFK2, thus defining a functional role for this lncRNA in metabolic reprogramming in breast cancer (91).

In malignant melanoma, expression of the lncRNA H19 was shown to be increased in melanoma tissue and might function as a sponge of miR-106a-5p to upregulate E2F3 expression and consequently promote the glucose metabolism and growth of melanoma cells (125).

Finally, in glioblastoma, the lncRNA XIST was revealed to regulate glucose metabolism by targeting GLUT1 and GLUT3 through the miR-126-dependent insulin receptor substrate 1 (IRS1)/PI3K/Akt pathway and to positively control glioblastoma cell growth *in vivo* and *in vitro* (117).

### Clinical Significance of lncRNAs in Cancer

Based on the above analysis, such lncRNAs involved in the regulation of cancers through glucose metabolism could be critical prognostic biomarkers or therapeutic targets. For example, Luan et al. reported that the level of the lncRNA H19 was increased in melanoma tissue and was correlated with poor prognosis of melanoma patients (125). In addition, LINP1 was found to promote the malignant phenotype of AML cells and to stimulate glucose metabolism, which was significantly upregulated in AML patients at diagnosis, but downregulated after complete remission (CR) (138). Moreover, UCA1 expression was found to be upregulated following ADR-based chemotherapy and its knockdown could overcome the chemoresistance of pediatric AML, indicating that it is to reasonably predictive of drug benefit (122). Moreover, in lung cancer, antisense oligonucleotides (ASO) blocking of MALAT1 was shown to prevent the development of metastasis *in vivo* (139, 140).

### Effect, Mechanism of Action, and Clinical Significance of lncRNAs in Metabolic Disease

#### Diabetes Mellitus

Diabetes mellitus (DM) is a metabolic disorder that is characterized by abnormal glucose metabolism, including hyperglycemia, in fasting and postprandial states. Several lncRNAs have been shown to regulate glucose metabolism and be associated with the pathology of type 2 DM (T2DM) (38, 53), involving changes in the growth of pancreatic β-cell, insulin synthesis, insulin secretion, and insulin signaling in target tissues (including liver, skeletal muscle, and adipocyte). For instance, Akerman et al. have demonstrated that the lncRNA PLUTO controlled 3D chromatin structure and the transcription of PDX1, a master regulator of pancreas development and β-cell differentiation, and that both PLUTO and PDX1 were downregulated in islets from organ donors with type 2 diabetes or impaired glucose tolerance, suggesting a potential role in DM (141). The lncRNA Meg3 has been demonstrated to affect the process of insulin biogenesis and be involved in the development of diabetes by inhibiting the expression of Rad21, Smc3, or Sin3α via EZH2-driven H3K27 methylation (29). In another study, Fadista et al. reported that the lncRNA LOC283177 was co-expressed with master genes of islet function (MADD, PAX6, and SYT11). Meanwhile, its expression was positively associated with insulin exocytosis, but negatively with HbA1c levels. However, its function remained unknown (92). Dysregulation of insulin signaling was another common underlying mechanism of T2DM. Several lncRNAs have been emerged as regulators of insulin signaling (43, 44, 142). As mentioned earlier, the hepatic lncRNA H19 has been identified as the most significantly downregulated lncRNA in the livers of db/db mice (43). Inhibition of hepatic H19 impaired insulin signaling and increased the expression of gluconeogenic genes, possibly by regulating FoxO1 nuclear levels (43). In contrast to the results of that study, Zhang et al. showed that H19 expression was chronically increased in diet-induced diabetic mouse liver and that liver-specific H19 overexpression promoted hyperglycemia and insulin resistance. At the molecular level, they demonstrated that H19-induced alteration in promoter methylation and expression of Hnf4a may contribute to the underlying mechanism (44). Additionally, skeletal muscle H19 can also regulate insulin signaling. Gao et al. reported that H19 was significantly decreased in muscle of T2DM patients and insulin-resistant rodents. Mechanistically, H19 could act as a molecular sponge to inhibit let-7 miRNA, this reduction led to increased bioavailability of let-7, causing reduced expression of let-7 targets, eventually resulting in impaired insulin sensitivity and increased blood glucose level (142).

Diabetes mellitus and its complications are major health concerns that can be mitigated by implementing early preventive measures in people who are at high risk. However, current risk models perform suboptimally with regard to predicting individual diabetes risk (143). As mentioned above, several lncRNAs involved in the pathogenesis of T2DM may serve as diagnostic biomarkers or therapeutic targets for T2DM. Saeidi et al. studied 100 T2DM patients and 100 non-diabetic controls and reported that the lncRNAs LY86-AS1 and HCG27_201 were downregulated in peripheral blood mononuclear cells (PBMCs) in T2DM patients compared with levels in a control group. They also found that the expression of both of these lncRNAs was negatively correlated with fasting blood sugar levels (144). In addition, Sathishkumar et al. reported a significant increase in the expression of several lncRNAs (e.g., HOTAIR, Meg3, LET, MALAT1, MIAT, CDKN2BAS1/ANRIL, XIST, PANDA, GAS5, Linc-p21, ENST00000550337.1, PLUTO, and NBR2) and a significant decrease in the expression of lncRNAs THRIL and SALRNA1 in 30 patients with T2DM compared with the levels in 30 control subjects (145). A recent meta-analysis by Zhang et al. also revealed that abnormal lncRNA expression had diagnostic value in T2DM patients. As diagnostic biomarkers, lncRNAs showed relatively high diagnostic accuracy for T2DM and prediabetes, with sensitivity of 0.71 and 0.76, respectively (146). Moreover, it has been asserted that the pathogenesis of gestational diabetes mellitus (GDM) is similar to that of T2DM (147). Several studies have shown that lncRNAs such as PVT1, MALAT1, and AC092159.2, could be novel biomarkers to predict and diagnose GDM, although the molecular mechanisms involved were unclear (148–150). These effects demonstrate the potentially vital roles of lncRNAs in DM and provide evidence that lncRNAs may serve as clinical risk and diagnostic biomarkers of T2DM. However, research on lncRNAs as possible therapeutic targets for T2DM has been scarce. High-yield extracellular vesicle-mimetic nanovesicle contained lncRNA H19 was considered an effective treatment that accelerated the healing process in diabetes-associated chronic wounds (151).

#### Obesity

Obesity has reached epidemic proportions worldwide and is an important risk factor for T2DM, hypertension, cardiovascular disease, and certain cancers. However, effective treatments are limited. The lncRNA-mediated dysfunction of glucose and lipid metabolism, especially their effects on adipocytes, has been closely related to obesity. LncRNA HOXA1-AS1 expression levels were positively correlated with adipogenesis, and its expression was significantly higher in obese patients than in controls (152). Knockdown of the lncRNA HOXA11-AS1 using siRNA attenuated adipocyte differentiation, leading to suppression the transcription of adipogenic genes, such as CEBP-α, DGAT2, CIDEC, and perilipin (152). Another lncRNA, named AC092159.2, was shown to be positively associated with body mass index (BMI) and obesity. Its overexpression promoted adipocytes differentiation while its knockdown led to an adipogenic defect. Mechanistically, this lncRNA enhanced human adipocytes differentiation, possibly by regulating TMEM18 (153). Conversely, obese mice were shown to have low lnc-U90926 expression in subcutaneous and visceral adipose tissue. The overexpression of lnc-U90926 attenuated 3T3-L1 adipocyte differentiation via inhibiting the transactivation of PPARγ2 or PPARγ (101). Increasing BAT thermogenesis in mice and humans can improve obesity. In one study, it was observed that the lncRNA H19 was related to brown adipocyte differentiation and was decreased in obesity in BAT under an obese state. H19 overexpression promoted while knockdown of H19 impaired adipogenesis in brown adipocytes through recruiting PEG (80). In addition, H19 transgenic mice were shown to be protected from diet-induced obesity (80). Moreover, SREBP-1c has been shown to be a critical transcription factor in FA synthesis (88). SREBP-1c expression was increased in the liver of obese patients (154). Finally, MALAT1 may induce lipid accumulation and insulin resistance by increasing SREBP-1c and target gene expression (59).

Although the precise molecular network that regulates obesity remained unclear, such lncRNAs may be involved in obesity and could be novel therapeutic targets for this condition. The use of synthetic molecules or bioactive compounds that mimic or inhibit specific lncRNAs or modulate the expression of specific lncRNAs through the application of siRNA and antisense oligos, as well as applying the powerful CRISPR-Cas9 gene editing approach, may become new molecular treatment strategies.

#### Atherosclerosis

Atherosclerosis is a leading cause of mortality from cardiovascular disease. The dysfunction of lipid metabolism is well-known to be a significant risk factor for atherosclerosis. Lipid disorders cause lipoprotein accumulation within middle and large artery walls, thus triggering atherosclerosis (155). Several lncRNAs are involved in lipid metabolism-related disorders through the regulation of lipid synthesis, reverse cholesterol transport, and bile acid excretion (63, 66). The formation of macrophage foam cells, which play central roles in the initiation and progression of atherosclerotic plaques, has been shown to be closely related to DYNLRB2-2, MeXis, and RP5-833A20.1 (63, 64, 67). Mechanistically, lincRNA-DYNLRB2-2 expression promoted ABCA1-mediated cholesterol efflux and inhibited inflammation through GPR119 in THP-1 macrophage-derived foam cells (63). MeXis increased ABCA1 expression in macrophages through interacting with and guiding promoter binding of DDX17 (124). RP5-833A20.1 downregulated NFIA in human foam-cell macrophages by modulating miR-382 levels (67). In addition, Meng et al. found that GAS5 deletion inhibited the progression of atherosclerosis by reducing EZH2-mediated ABCA1 transcription in ApoE knockout mice (66).

The lncRNA MALAT1 in peripheral blood cells has been implicated in left ventricular dysfunction in patients with myocardial infarction (MI) (156) and might be associated with lipid accumulation and insulin resistance (59). Indeed, atherosclerotic burden was significantly reduced in Ldlr knockout animals by treatment with an AAV8 vector expressing LeXis (157). At the molecular level, lncRNA LeXis overexpression improved atherosclerosis through reducing the expression of SREBF2 and HMGCR via preventing the Raly-mediated recruitment of RNA polymerase II (55). These findings demonstrate the feasibility of using lncRNAs as predictors and diagnostic markers of atherosclerosis and in mimetic therapeutic strategies. However, further studies are needed to explore the detailed molecular mechanisms of lncRNAs so that target gene therapy may proceed to clinical trials.

#### NAFLD

NAFLD is a common metabolic disorder of the liver that has emerged as a major public health problem with a high prevalence rate (12.9–46.0% in various countries) (158). An increasing number of studies have explored the pathogenesis of NAFLD (159, 160), but effective strategies for preventing and treating NAFLD are still lacking. The accumulation of triglycerides within hepatocytes is a hallmark of NAFLD. Emerging evidence shows that some lncRNAs regulate triglyceride metabolism in hepatocytes and in a mouse model of NAFLD by controlling triglyceride biosynthesis and CD36-dependent lipid uptake (59, 93, 161–163). In the liver of obese mice and patients with NAFLD, the expression of mouse lncSHGL and its human homolog lncRNA B4GALT1-AS1 was found to be decreased (161). Hepatic lncSHGL restoration reduced triglyceride content and improved hyperglycemia, insulin resistance, and steatosis in obese diabetic mice by activating the PI3K/Akt pathway and repressing the mammalian/mTOR/SREBP-1c pathway independent of insulin and calcium (161). Similarly, the lncRNA MALAT1 promoted hepatic steatosis and insulin resistance by increasing nuclear SREBP-1c stability (59). uc.372 expression was also shown to increase in the liver in db/db mice, high-fat-diet-fed mice, and NAFLD patients through regulating CD36-dependent lipid uptake (93). Another study has showed that mice with knockout of the lncRNA SRA had decreased hepatic triglyceride content. Further experiments elucidated that SRA could inhibit the expression of adipose triglyceride lipase (ATGL), which is a key lipolytic enzyme, by repressing the transcriptional activity of FoxO1 (162). Moreover, the lncRNA MEG3 was significantly decreased in liver tissues of NAFLD patients (164). Mechanistically, Huang et al. demonstrated that MEG3 could act as a ceRNA for miR-21 to regulate LRP6 expression during liver lipid deposition (156).

Sun et al. performed a genome-wide analysis and found the aberrant expression of lncRNAs in liver tissue in NAFLD patients. A total of 1735 lncRNAs were differentially expressed in NAFLD samples compared with control samples (164). Di Mauro et al. also performed a genome-wide analysis and found that lncRNA RP11-128N14.5 expression was upregulated in liver tissue and serum in severe NAFLD patients compared with mild NAFLD patients and controls (165). These findings clearly suggest that lncRNAs may serve as non-invasive diagnostic markers and novel therapeutic targets for NAFLD in the future.

## Conclusions and Further Prospects

lncRNAs have broad implications in glucose and lipid metabolism in physiological and pathological conditions. They mediate various biological processes in different tissues and diseases. The lncRNAs involving in regulating glucose metabolism mainly participated in the development of pancreatic islet cells, insulin biogenesis, insulin secretion, and manipulate insulin signaling in peripheral tissues (including liver, skeletal muscles, and adipocytes). In addition, lncRNAs act in complex processes to regulate lipid metabolism via cholesterol and triglyceride biosynthesis by regulating expression of the transcription factor SREBP, lipid efflux by regulating ABCA1 expression and lipoprotein formation, LDLR- and CD36-dependent lipid uptake, bile acid metabolism, and adipogenesis. In pathological conditions, abnormal lncRNAs could participate in the regulation of various cancers and contribute to metabolic diseases, including T2DM, obesity, cardiovascular disease, and NAFLD through influencing glucose and lipid metabolism.

The diversity of responses that are observed in different tissues and diseases demonstrates the complex functions of lncRNAs in the body. This review should help to provide a better understanding of lncRNAs in glucose and lipid metabolism, potentially allowing better predictions of disease states and contributing to novel therapeutic strategies that exploit relevant signaling pathways. The studies described here have provided strong evidence that lncRNAs play vital roles in glucose and lipid metabolism, but a number of important questions remain unanswered. First, the precise molecular mechanism in different pathological conditions is still unclear. In addition, the expression levels of many lncRNAs are quite low, which brings some uncertainty regarding the reliability and reproducibility of large-scale lncRNA investigations. There are still other limitations associated with using circulating or tissue lncRNAs as biomarkers, difficulty measuring lncRNAs, low accuracy, and expensive procedures. Moreover, the poor DNA sequence conservation of lncRNAs across species can be an issue for the extrapolation of research results obtained from animal models to humans. For the translation of lncRNA-based therapy into clinical applications, the establishment of a better delivery system should also be considered. Therefore, future work on the involvement of lncRNAs in glucose and lipid metabolism might include the following:

1) Effect of crosstalk of exosome-derived lncRNAs among the tissues.
2) A thorough functional characterization of lncRNAs in glucose and lipid metabolism-related disease, at both the molecular and the cellular levels.
3) Retrieving the most promising candidates for therapeutic targets from the huge amount of sequencing data available.
4) Identifying panels of lncRNAs for optimal accuracy for diagnosing glucose and lipid metabolism-related disease.
5) Exploring glucose and lipid metabolism-related disease risk reduction models using lncRNA biology.
6) Carrying out more research on lncRNA-based therapy *in vivo* using optimal delivery systems.

## Author Contributions

All authors listed have made a substantial, direct and intellectual contribution to the work, and approved it for publication.

## Conflict of Interest

The authors declare that the research was conducted in the absence of any commercial or financial relationships that could be construed as a potential conflict of interest.
